# Effects of Mixed Culture Fermentation on Quality of Non-Fried Whole-Wheat Instant Noodles

**DOI:** 10.3390/foods15132265

**Published:** 2026-06-24

**Authors:** Hao-Ran Han, Rui-Xian Shang, Wan-Wan Cui, Yun Chen, Lin-Lin Li, Xiao-Ling Tian, Jian Zhang, Yang Zhao

**Affiliations:** 1 College of Food Science and Technology, Henan Agricultural University, Zhengzhou 450002, China; hanhaoran0830@163.com (H.-R.H.); 2775785693@qq.com (R.-X.S.); sweetc0509@163.com (Y.C.); linl@henau.edu.cn (L.-L.L.); ttxling@henau.edu.cn (X.-L.T.); 2 Baixiang Foods Co., Ltd., Zhengzhou 451100, China; cuiwwwise@126.com; 3 Key Laboratory of Staple Grain Processing, Ministry of Agriculture and Village, Zhengzhou 450002, China

**Keywords:** mixed culture fermentation, non-fried whole-wheat instant noodles, volatile compounds, in vitro digestibility

## Abstract

Non-fried whole-wheat instant noodles feature high dietary fiber and balanced nutrition but suffer from poor rehydration, texture, and flavor. This study aims to improve the quality of these noodles through mixed fermentation of yeast and lactic acid bacteria (LAB). The rehydration characteristics, textural profile, sensory quality, microstructure, volatile flavor compounds, and in vitro digestibility of non-fried instant whole-wheat noodles were evaluated. Yeast primarily reduced rehydration time and improved mouthfeel, whereas LAB mainly contributed to the formation of a continuous and uniform gluten network as well as the enhancement of noodle flavor. Moderate addition of the mixed culture (1% yeast + 0.05% LAB) promoted the construction of a homogeneous gluten network in non-fried whole-wheat instant noodles, effectively reducing starch digestibility and estimated glycemic index (eGI). However, excessive addition caused opposite effects on these noodles. This study provides an effective processing strategy for the production of high-quality, low-eGI whole-wheat instant noodles, which are suitable for people pursuing healthy diets and controlling blood sugar levels.

## 1. Introduction

Instant noodles are globally recognized and consumed for their palatability, convenience, and affordability [1]. Meanwhile, rising consumer preference for healthy diets has boosted the development of low-fat and non-fried functional instant noodles. Different from traditional fried counterparts, such products adopt hot air, microwave, or infrared drying technologies [2]. These drying methods effectively lower fat content and mitigate the formation of harmful compounds like saturated and trans fats typically introduced during frying [3]. Conventional instant noodles are mainly manufactured from refined wheat flour, which is high in carbohydrate and fat content, while lacking multiple essential nutrients required for daily dietary intake. Therefore, it is of great necessity to develop non-fried instant noodles with enhanced nutritional value [3].

Whole-wheat flour has attracted widespread research interest, owing to its ability to improve the nutritional profile of food products and retard starch digestion, which contributes to a reduced glycemic index [4]. In comparison with refined wheat flour, whole-wheat flour is richer in dietary fiber, B vitamins, lutein and β-carotene, as well as mineral substances including magnesium and selenium. It also contains abundant essential amino acids and bioactive phytochemicals such as polyphenols, flavonoids, and plant sterols [5]. Furthermore, the bran component provides additional dietary fiber, which can promote the formation of resistant starch (RS), helping modulate glycemic response and increase satiety [6]. Thus, whole-wheat flour was selected as the main ingredient for non-fried instant noodles in this study.

However, non-fried whole-wheat instant noodles still exhibit drawbacks, including poor rehydration properties and limited flavor profiles inherent to non-fried instant noodles [7], as well as undesirable sensory attributes of whole-wheat products such as dull dark color, mottled surface appearance, rough and firm texture, sour and bitter taste, alongside malty or musty off-flavors [8]. In addition, the abundant bran particles and dietary fiber in whole-wheat flour exert markedly negative effects on the dough system. On the one hand, bran particles possess sharp edges and irregular morphologies. They can physically disrupt and fracture the gluten network structure, impede the full hydration and cross-linking of gluten proteins, and ultimately weaken the integrity and continuity of the gluten matrix [9]. On the other hand, high levels of dietary fiber (especially insoluble dietary fiber) competitively absorb water and lead to inadequate hydration of gluten proteins. Meanwhile, their physical barrier effect also constrains starch granule swelling and gelatinization, ultimately resulting in hardened texture, reduced elasticity, and rough mouthfeel of whole-wheat noodles [10].

Compared to physical treatments (vacuum freeze-drying, convection drying) and chemical additives (guar gum, distilled monoglycerides) [11], co-fermentation technology is considered an effective, safe, and eco-friendly approach to solve the above-mentioned drawbacks. Our team has previously demonstrated that mixed culture fermentation using yeast and lactic acid bacteria (LAB) significantly improves the quality of non-fried instant noodles [12]. Firstly, this technique can modify the texture of flour products and physicochemical properties of starch [13,14]. For instance, pre-fermentation with yeast and LAB in brown rice noodle systems greatly elevated starch molecular order, further optimizing the hardness, elasticity, and chewiness of the final noodles [15]. Secondly, both microorganisms play crucial roles in flavor development. Their synergistic fermentation enriches the volatile compound profile and diversifies the aroma of the flour products [16,17,18]. Additionally, Vernon-Carter et al. [19] have found that yeast fermentation can reduce rapidly digestible starch (RDS) and increase RS in corn tortillas, providing a viable strategy for producing low-eGI products. Therefore, mixed culture fermentation using yeast and LAB may prove beneficial for improving noodle product quality, which can be demonstrated by measuring its effects on noodles’ physicochemical properties, flavor profile, and in vitro digestibility.

This study used whole-wheat flour as the main ingredient and applied a mixed fermentation technique with yeast and LAB. The effects of culture addition on rehydration characteristics, texture, microstructure, sensory quality, volatile components, and in vitro starch digestibility of non-fried whole-wheat instant noodles were investigated. The relationship between starch digestibility and noodle microstructure was also examined. This research supports the application of mixed culture fermentation technology in non-fried instant noodle production, catering to the growing demand for healthy and low estimated glycemic index (eGI) staple food.

## 2. Materials and Methods

### 2.1. Materials

Whole-wheat flour was purchased from Jinyuan Flour Co., Ltd. (Zhengzhou, China), with 24.00% protein, 2.33% ash, and 7.20% moisture (*w*/*w*). Gluten flour was provided by Feitian Biotech Co., Ltd. (Hebi, China), with 84.30% protein, 8.37% ash, and 9.70% moisture (*w*/*w*). Active dry yeast was supplied by Angel Yeast Co., Ltd. (Yichang, China), with a viable count of 3 × 10^10^ CFU/g. LAB (strain YC-381) was provided by Chr. Hansen Trading Co., Ltd. (Beijing, China). Edible alkali was purchased from a local supermarket. Amyloglucosidase (A7095, ≥260 U/mL), pancreatin from porcine pancreas (P7545, 8 × USP), and pepsin (P7000, ≥250 U/mg) were all purchased from Sigma-Aldrich (St. Louis, MO, USA). The GOPOD kit was supplied by Megazyme International Ireland (Bray Business Park, Bray, Co. Wicklow, Ireland). All other chemical reagents used in this study were of analytical grade.

### 2.2. Preparation of Non-Fried Whole-Wheat Instant Noodles

A base mixed flour was prepared with 255 g of whole-wheat flour, 45 g of gluten, and 0.6 g of edible alkali. The mixed culture (yeast-LAB) was activated in 108 g of water (adjusting the total moisture content of the noodles to 36%) and then added to the base mixed flour (*w*/*w* of total flour).

To explore the effect of mixed culture addition on the quality of non-fried whole-wheat instant noodles, this study employed mixed fermentation with a fixed ratio of yeast and LAB. Based on preliminary experiments regarding culture ratios (set at 0, 100:1, 50:1, 20:1, 10:1, and 5:1), a ratio of 20:1 was determined as optimal, as it contributed to the shortest rehydration time, optimal textural properties, and the highest sensory scores for the noodles. Furthermore, referencing our previous studies on cereal fermentation [12,20] and preliminary experimental results, the concentration gradient for yeast was established at 0–2.5%. Consequently, the experimental groups were designed as follows: with the fixed yeast-to-LAB ratio of 20:1, the total mixed culture addition was set as a gradient. Yeast was added at 0.5%, 1.0%, 1.5%, 2.0% and 2.5%, corresponding to LAB addition of 0.025%, 0.05%, 0.075%, 0.1% and 0.125%, respectively, denoted as Y0.5-L0.025, Y1.0-L0.05, Y1.5-L0.075, Y2.0-L0.1, and Y2.5-L0.125. With yeast addition fixed at 1.0%, the effect of LAB gradient addition was explored. LAB was supplemented at 0%, 0.01% and 0.1%, marked as Y1.0-L0, Y1.0-L0.01, and Y1.0-L0.1. The control group was set as Y0-L0 [12].

The flour mixture was blended with the activated bacterial suspension in a ZNH vacuum dough mixer (Jiangsu Dazhong Transmission Equipment Co., Ltd., Taizhou, China) for 12 min.

Referring to the two-stage fermentation process for non-fried instant noodles reported by Zhang et al. [12], this fermentation duration was sufficient to enable full metabolic activity of the mixed culture, which effectively improves the rehydration property and flavor quality of final products. The dough was fermented at 30 °C for 30 min, then sheeted through a JMTD168 noodle sheeter (Beijing Dongfu Jiuheng Instrument Technology Co., Ltd., Beijing, China), and cut into noodles with a width of 0.8 mm. The non-fried whole-wheat instant noodles were obtained after the noodles underwent a fermentation at 30 °C for another 30 min, steaming for 5 min, and drying at 80 °C for 90 min.

### 2.3. Rehydration Characteristics

The optimal rehydration time was determined according to the method described by Ding et al. [21]. Noodle strands were completely submerged in boiling water within an insulated container. One noodle was removed every 10 s and pressed between glass plates to check for the hard core. The time at which no hard core remained was recorded as the optimal rehydration time.

Cooking tolerance was assessed using the method described by Zhang et al. [12]. Noodle hardness was measured after rehydration in boiling water for 10 min.

### 2.4. Textural Properties

Textural properties were measured by a TA-XA Texture Analyzer (Stable Micro Systems Ltd., Godalming, UK) following the method of Yahata et al. [22] with minor modifications.

TPA: Three noodle strands, cooked to their optimal rehydration time, were placed parallel and equidistantly on the platform. Each group was measured in six replicates. Parameters were set as follows: pre-test speed, 2 mm/s; test speed, 1 mm/s; post-test speed, 2 mm/s; trigger force, 5 g.

Shear test: For each group (6 replicates), 3 rehydrated noodle strands were arranged perpendicular to the shear blade. Parameters were set as follows: pre-test speed, 2 mm/s; test speed, 0.8 mm/s; post-test speed, 0.8 mm/s; trigger force, 3 g.

Tensile test: One single rehydrated noodle strand was attached to the tensile grips. The upper grip was raised at a constant speed until the strand broke. Parameters were set as follows: pre-test speed, 2 mm/s; test speed, 2 mm/s; post-test speed, 10 mm/s; trigger force, 5 g.

For all tests (TPA, shear, tensile), noodles were prepared by cooking to the optimum rehydration time.

### 2.5. Scanning Electron Microscope (SEM) Analysis

The microstructure and morphology of the instant noodles were observed using a scanning electron microscope (Q45, Thermo Fisher Scientific, Waltham, MA, USA) according to the method described by Pinyo et al. [23] with slight modifications. Cross-sections and cut surfaces of the samples were mounted on the stubs using conductive adhesive tape, sputter-coated with gold for 35 s, and then examined. SEM images were captured at magnifications of 500× and 1000×, respectively.

### 2.6. Fourier Transform Infrared Spectra (FTIR) Analysis

FTIR spectroscopic analysis was carried out following the protocol described by Ge et al. [17] with minor modifications. The instant noodle samples were fully crushed and screened through an 80-mesh sieve to obtain uniform fine powder. The obtained sample powder was blended with potassium bromide (KBr) at a mass ratio of 1:100, followed by thorough grinding and tabletting to form uniform sample pellets. Spectral data were acquired using a Nicolet 6700 FTIR spectrometer (Thermo Fisher Scientific, Waltham, MA, USA). The scanning wavenumber range was set from 4000 cm^−1^ to 400 cm^−1^, with 32 cumulative scanning times and a spectral resolution of 4 cm^−1^. Spectral preprocessing operations, including baseline correction, Gaussian deconvolution, and second derivative analysis, were carried out via OMNIC 9.0 software (Thermo Fisher Scientific). Based on reported conventional methods, PeakFit 4.12 software (Systat Software, San Jose, CA, USA) was adopted for the quantitative analysis of protein secondary structure. The second-derivative spectrum of the amide I band within 1600–1700 cm^−1^ was fitted using Gaussian functions for structural quantification.

### 2.7. X-Ray Diffraction (XRD) Analysis

XRD analysis was performed using an X-ray diffractometer (Bruker AXS Inc., Karlsruhe, Germany) following a modified method from Zhang et al. [24]. Samples were ground and sieved through an 80-mesh sieve. The sieved powder was densely packed into a glass sample holder and placed on the instrument stage. Measurements were conducted using Cu-Kα radiation at 30 kV, scanning over a diffraction angle (2θ) range of 4° to 40° at a rate of 2°/min and a step width of 0.02°. Calculation of sample relative crystallinity was performed with the support of Origin 2024 analytical software (OriginLab Corporation, Northampton, MA, USA).

### 2.8. Determination of Volatile Compounds

Volatile components of the five selected noodle samples were analyzed using GC-MS (ISQ7000, Thermos Scientific, Waltham, MA, USA) based on a method described by Shang et al. [20] with minor modifications.

Briefly, 3 g of pulverized instant noodle sample was placed into a 20 mL headspace vial. A total of 2 μL of 0.20 mg/mL 2-octanol solution was added as the internal standard, and the vial was tightly sealed with a PTFE/silicone rubber septum. The vial was incubated in a water bath at 60 °C for 10 min to achieve equilibrium. A pre-aged 50/30 μm DVB/CAR/PDMS SPME fiber was then exposed to the headspace of the vial for 50 min of volatile compound extraction. Afterwards, the extraction fiber was desorbed in the GC inlet for 2 min before instrumental detection.

Separation was performed on an HP-5 MS capillary column (30 m × 0.25 mm × 0.25 μm). The oven temperature program was: Initially stay at 40 °C for 2 min, rise to 70 °C at 10 °C/min and maintain for 2 min; increase to 170 °C at 3 °C/min and hold for 2 min; finally, increase to 210 °C at 8 °C/min and hold for 2 min. High-purity helium (99.9999%) was used as the carrier gas at a constant flow rate of 1.0 mL/min. MS parameters were: ion source temperature, 230 °C; electron impact energy of 70 eV; scan mode, full scan (mass range 35–500 *m*/*z*).

Qualitative analysis: Volatile compound peaks obtained from mass spectrometry were matched against the NIST17 spectral database (National Institute of Standards and Technology, Gaithersburg, MD, USA). Only compounds with a match factor ≥ 800 were retained. Quantitative analysis: The internal standard method with 2-octanol as the reference substance was adopted for quantitative determination, and the content of each identified compound was calculated according to the following formula:

(1) $$M_{x}=\frac{2\times S_{x}\times 1000}{3\times S_{A}\times 1000}\times 200$$

where $S_{x}$ and $S_{A}$ are the peak areas of the target compound and the internal standard, respectively; 2 is the addition of the internal standard, μL; 3 is the mass of the sample, g. $M_{x}$ is the content of the target compound, μg/kg.

Each treatment was prepared in triplicate with good repeatability, and one representative sample was selected for GC-MS analysis.

### 2.9. In Vitro Digestibility Properties

The in vitro starch digestibility was evaluated based on the method of Englyst et al. [25] with modifications. After rehydration to optimal condition, the 0.5 g noodle sample was mixed with 10 mL of distilled water in a 50 mL centrifuge tube, followed by centrifugation at 4500 r/min for 10 min. Following the removal of the supernatant, 5 mL of pepsin solution was introduced, and the mixture underwent shaking in a constant-temperature water bath for 30 min. Afterward, the sequential addition of 5 mL of 0.1 mol/L sodium hydroxide solution, 15 mL of 0.1 mol/L phosphate buffer, 5 mL of α-amylase, 1 mL of amyloglucosidase, and 1 mL of pancreatin was performed, followed by incubation with shaking at 37 °C for 180 min.

Aliquots of 0.1 mL digestive solution were collected at 0, 20, 60, 120, 150, and 180 min, and immediately blended with 0.7 mL absolute ethanol to terminate the reaction. The mixture was centrifuged at 1000 r/min for 10 min, after which 0.1 mL supernatant was added to 3.0 mL GOPOD reagent and incubated in a water bath at 50 °C for 20 min. Absorbance was measured at 510 nm.

The glucose content was determined according to a glucose standard curve. The starch hydrolysis degree was calculated to establish the corresponding hydrolysis profile, followed by the determination of eGI values. Experimental data were fitted using the nonlinear kinetic model: $C=C_{\infty }\left(1-e^{-kt}\right)$. In this equation, *C* represents the starch hydrolysis rate at time *t*, $C_{\infty }$ refers to the equilibrium hydrolysis percentage, and *k* denotes the kinetic rate constant. *RDS*, slowly digestible starch (*SDS*), and *RS* were calculated using the following equations:

(2) $$RDS\left(\%\right)=\left(G_{20}-G_{0}\right)\times 0.9\times 100$$

(3) $$SDS\left(\%\right)=\left(G_{120}-G_{20}\right)\times 0.9\times 100$$

(4) $$RS\left(\%\right)=100\%-RDS-SDS$$

### 2.10. Sensory Evaluation

Sensory assessment was performed by 20 trained and screened panelists aged 20–40 years, with an equal gender ratio of 10 males and 10 females. All samples were coded with random three-digit numbers for blind evaluation and served to assessors in a randomized sequence. The evaluated sensory indices covered color, appearance, hardness, stickiness, smoothness, taste, and flavor, with detailed scoring standards listed in Table S1 in the Supplementary Materials. Commercial mineral water (C’estbon, China Resources C’estbon Beverage Co. Ltd., Shenzhen, China) was supplied for mouth rinsing between sample evaluations.

### 2.11. Data Analysis

All experimental treatments were performed in triplicate, and all data were expressed as the mean ± standard deviation. Analysis of variance (ANOVA) was conducted using the SPSS 27.0 software (IBM Corp., Armonk, NY, USA), and significant differences among means were identified by Duncan’s test at *p* < 0.05 level. All figures in this study were plotted with Origin 2024 software (OriginLab Corporation, Northampton, MA, USA).

## 3. Results and Discussion

### 3.1. Effect of Mixed Culture Addition on Rehydration Characteristics of Non-Fried Whole-Wheat Instant Noodles

**Figure 1:**
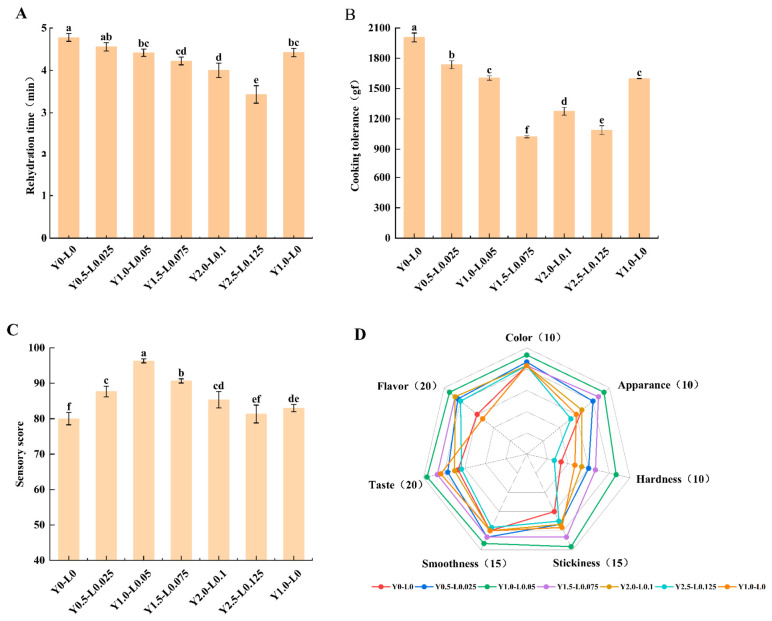
Effect of mixed culture addition on rehydration characteristics and sensory properties of non-fried whole-wheat instant noodles. (**A**) Rehydration time; (**B**) cooking tolerance; (**C**) sensory score; (**D**) sensory attribute radar chart. Samples tested were of control group (Y0-L0), 0.5% yeast with 0.025% lactic acid bacteria (LAB) (Y0.5-L0.025), 1.0% yeast with 0.05% LAB (Y1.0-L0.05), 1.5% yeast with 0.075% LAB (Y1.5-L0.075), 2.0% yeast with 0.1% LAB (Y2.0-L0.1), 2.5% yeast with 0.125% LAB (Y2.5-L0.125), and 1.0% yeast with no LAB (Y1.0-L0). Bars with different letters indicate statistically significant differences between each other (*p* < 0.05).

Rehydration time and cooking tolerance are crucial quality indicators for instant noodles, with high-quality noodles featuring fast rehydration and high hardness after 10 min of rehydration. Whole-wheat flour contains abundant insoluble dietary fiber (predominantly bran cellulose and hemicellulose) with strong water absorption, forming a dense surface barrier that hinders internal water penetration and delays the rehydration of whole-wheat instant noodles [26]. However, the optimal rehydration time of non-fried whole-wheat instant noodles decreased significantly (*p* < 0.05) with varying levels of mixed culture addition (Figure 1A). This is attributed to the porous structure formed by carbon dioxide (CO_2_) produced from yeast metabolism of carbohydrates during fermentation [20]. Specifically, with the increase in mixed culture addition, the CO_2_ production of the dough gradually rises, increasing the number and pore size of voids within the gluten network. This loose and porous structure not only remarkably enlarges the specific surface area of noodles, but also provides pathways for water to bypass the barrier of bran particles, effectively overcoming the hindrance of dietary fiber on water diffusion and thereby markedly shortening rehydration time [7]. Additionally, the hardness of noodles after 10 min of rehydration also generally exhibited a decreasing trend with increasing culture addition, and the decline was particularly pronounced after Y1.0-L0.05 (Figure 1B).

Y1.0-L0.05 and Y1.0-L0 both showed a rehydration time of 4.4 min (264 s) and hardness values of 1605 gf and 1598 gf after 10 min rehydration, respectively (Figure 1B), exhibiting comparable rehydration characteristics and cooking tolerance. This indicates that yeast is the primary factor influencing the rehydration time and cooking tolerance of non-fried whole-wheat instant noodles, consistent with Zhang et al. [12].

On the whole, Y1.0-L0.05 exhibited a moderate rehydration time and favorable hardness after 10 min of rehydration. At this stage, the moderate yeast fermentation avoids the insufficient porosity and bran-induced rehydration retardation caused by under-fermentation, while mitigating gluten damage from excessive CO_2_ production during over-fermentation.

### 3.2. Effect of Mixed Culture Addition on the Textural Properties of Non-Fried Whole-Wheat Instant Noodles

As shown in Table 1, hardness and chewiness decreased with the addition of mixed culture. This reduction is due to the CO_2_ produced by yeast expanding the compact internal structure, and the organic acids generated by LAB, which soften bran and promote gluten protein hydrolysis and solubility [7,8]. Compared with Y0-L0, Y1.0-L0.05 exhibited decreased hardness (from 2393.31 gf to 1858.01 gf, 22.4% reduction) and chewiness (from 1568.95 gf to 1205.31 gf, 23.2% reduction). Notably, Y1.5-L0.075 showed substantial declines of 40.8% in hardness and 43.4% in chewiness, revealing 1% yeast combined with 0.05% LAB as the critical threshold for noodle texture.

Adhesiveness significantly reduced with the increasing mixed culture addition (*p* < 0.05), suggesting that fermentation effectively improved the sticky mouthfeel of whole-wheat noodles. This is because microbial metabolites degrade surface sticky starch, while LAB-generated organic acids suppress water absorption and adhesion of dietary fibers [7]. Springiness showed the same trend (*p* < 0.05), demonstrating that fermentation weakened the structural support of noodles.

Shear force, toughness, and tensile strength generally decreased with mixed culture addition, but rose slightly at Y2.0-L0.1. This may reflect metabolite accumulation inhibiting bacterial activity and reducing gluten structure degradation. Furthermore, organic acids and proteases from metabolism promoted mild gluten degradation and rearrangement, forming new protein cross-linked structures that repaired the loose dough matrix [20].

There was no significant difference between Y1.0-L0 and Y1.0-L0.01 in terms of hardness, adhesiveness, springiness, chewiness, or tensile properties, and only shear force and toughness were slightly reduced in Y1.0-L0.01. This demonstrates yeast as the primary factor affecting the textural properties of non-fried whole-wheat instant noodles.

In conclusion, Y1.0-L0.05 effectively improved the hardness and stickiness of whole-wheat noodles while maintaining good elasticity, achieving the best textural quality.

### 3.3. Effect of Mixed Culture Addition on Sensory Properties of Non-Fried Whole-Wheat Instant Noodles

As shown in Figure 1C, the sensory score of non-fried whole-wheat instant noodles first rose and then declined with the increase in mixed culture addition. Y1.0-L0.05 achieved the highest overall sensory score of 96 points, which was higher than the others.

Figure 1D indicates that Y0-L0 exhibited prominent undesirable sensory attributes, including inherent dark color, speckled appearance, rough and hard texture, as well as bitter, sour, malty, or musty flavors due to the absence of fermentation [8]. Y0.5-L0.025 only partially improved the sensory quality, and the rough mouthfeel caused by insufficient fermentation was not fully eliminated. By contrast, Y2.5-L0.125 produced excessive organic acids via over-fermentation, resulting in an unpleasant, overly sour flavor and disruption of the gluten network structure. This led to increased adhesiveness and unbalanced hardness, ultimately reducing the overall sensory score. Moreover, Y1.0-L0 showed distinctly inferior sensory performance compared with the others, demonstrating that synergistic fermentation of yeast and LAB could alleviate the roughness and astringency of bran fibers, mask the bitter taste of bran, and comprehensively enhance the sensory acceptability of non-fried whole-wheat instant noodles.

Based on the above results, Y1.0-L0.05 presented moderate rehydration time, good cooking tolerance, low viscosity, smooth mouthfeel, and medium-to-high hardness and chewiness with desirable softness and elasticity, along with the optimal sensory evaluation. Accordingly, the optimal compound bacteria addition was determined as 1% yeast with 0.05% LAB for non-fried whole-wheat instant noodles.

### 3.4. Effect of Mixed Culture Addition on Microstructure of Non-Fried Whole-Wheat Instant Noodles

**Figure 2:**
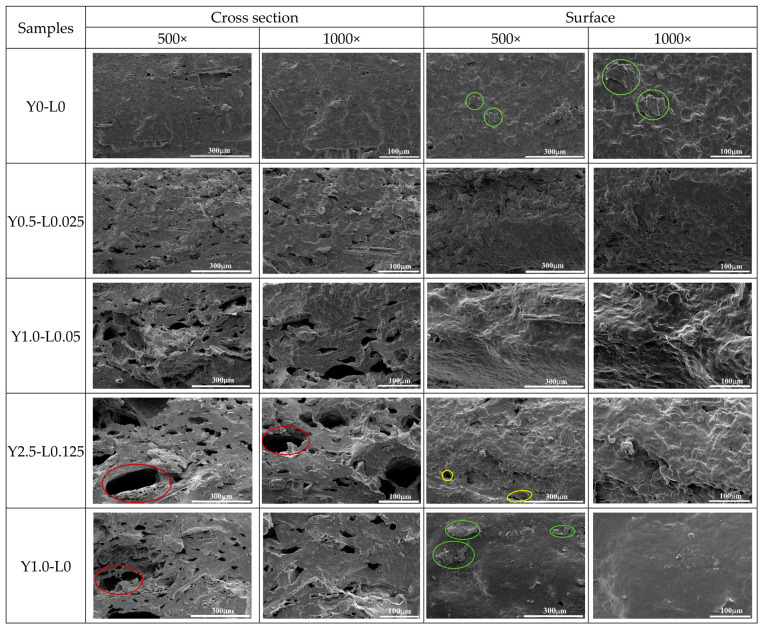
Effect of mixed culture addition on microstructure of non-fried whole-wheat instant noodles. Samples tested were of the control group (Y0-L0), 0.5% yeast with 0.025% lactic acid bacteria (LAB) (Y0.5-L0.025), 1.0% yeast with 0.05% LAB (Y1.0-L0.05), 2.5% yeast with 0.125% LAB (Y2.5-L0.125), and 1.0% yeast with no LAB (Y1.0-L0). The red circles represent the pores formed during the fermentation, the green circles mark the presence of brans, and the yellow circles indicate ruptured gluten networks.

SEM cross-sections (Figure 2) revealed a progressive increase in pore number and size with higher mixed culture levels. Y0-L0 exhibited an almost pore-free, compact internal structure, whereas Y0.5-L0.025 and Y1.0-L0.05 developed significantly more numerous, evenly distributed pores. This is because CO_2_ produced by yeast formed a loose and porous gluten network [27]. These uniform pores not only enhanced the water absorption capacity of noodles but also alleviated the mechanical stress on the gluten network induced by fiber expansion, thereby shortening the rehydration time and reducing the excessive hardness caused by the dense noodle matrix [7,28]. These findings were consistent with rehydration and texture results in Table 1. Conversely, Y2.5-L0.125 showed large, irregular, interconnected pores and severely fractured gluten networks, indicating that over-fermentation impaired the ability of gluten proteins to entrap gas, markedly reduced structural stability, and consequently caused noodle breakage and internal looseness. Notably, Y1.0-L0.05 possessed significantly more pores with more uniform pore size distribution than Y1.0-L0, indicating yeast primarily produced gas, while LAB remarkably improved gluten extensibility and gas retention [29].

Surface SEM images further revealed the stretched state of the gluten network and the distribution characteristics of starch granules. Compared with Y1.0-L0.05, Y1.0-L0 lacked distinct fibrous stretching textures, showing inadequately stretched restructured gluten networks and conspicuous bran particles. This suggests that LAB may contribute to gluten network extension and bran softening. This could be attributed to organic acids produced by LAB, which activated endogenous xylanase activity and promoted the cleavage of bran dietary fiber, thereby softening bran and alleviating its destructive effect on the gluten network [14]. Moreover, in Y0-L0, the gluten networks were densely packed and tightly wrapped around starch granules, reflecting a rigid and compact structure of the unfermented noodles. Meanwhile, unsoftened bran fragments embedded in the matrix disrupted gluten continuity. In contrast, Y0.5-L0.025 exhibited a more uniform and smoother gluten network with fewer damaged starch granules. This further confirmed the crucial role of LAB in promoting gluten network extension and bran softening. Y2.5-L0.125 displayed severely stretched gluten networks with fractures and ruptures, alongside extensively exposed and loosely arranged starch granules. This facilitated the inward migration of amylases and organic acids, further accelerating starch hydrolysis and weakening the gluten network, and ultimately resulting in an excessively loose structure [30]. Compared with Y1.0-L0.05, Y1.0-L0 lacked distinct fibrous stretching textures, showing inadequately stretched restructured gluten networks and conspicuous bran particles, further confirming the crucial role of LAB in promoting gluten network extension and bran softening.

In summary, yeast mainly forms the porous structure through gas production, while LAB contributes to bran softening and the formation of a continuous and homogeneous gluten network. The synergistic effect of the two strains jointly optimizes the microscopic structure of noodles.

### 3.5. Effect of Mixed Culture Addition on FTIR and Protein Secondary Structure of Non-Fried Whole-Wheat Instant Noodles

**Figure 3:**
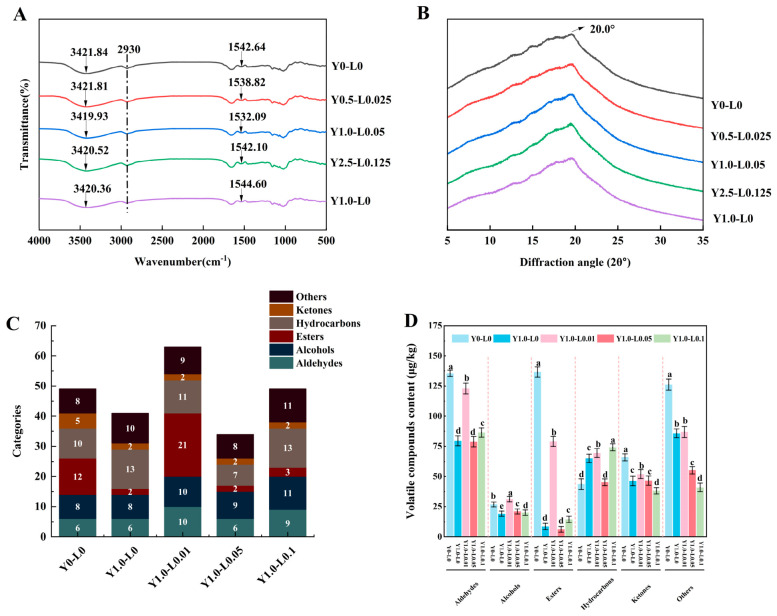
Effect of mixed culture addition on starch structure and volatile compounds of non-fried whole-wheat instant noodles. (**A**) Fourier-transform infrared spectroscopy, (**B**) XRD patterns, (**C**) number of volatile species, (**D**) volatile compounds content. Samples tested were of the control group (Y0-L0), 0.5% yeast with 0.025% lactic acid bacteria (LAB) (Y0.5-L0.025), 1.0% yeast with 0.05% LAB (Y1.0-L0.05), 2.5% yeast with 0.125% LAB (Y2.5-L0.125), 1.0% yeast with no LAB (Y1.0-L0), 1.0% yeast with 0.01% LAB (Y1.0-L0.01), and 1.0% yeast with 0.1% LAB (Y1.0-L0.1). Bars with different letters indicate statistically significant differences between each other (*p* < 0.05).

The FRIR spectra of noodles with different mixed culture additions are displayed in Figure 3A. All samples showed a strong, broad absorption band between 3000 and 3600 cm^−1^, attributed to O-H stretching vibrations and hydrogen bonds [13]. The peak at 2930 cm^−1^ corresponds to C-H stretching vibrations of saturated carbon [31]. The peak near 1538 cm^−1^ indicates the presence of starch-protein complex in the noodles [32]. Compared to Y0-L0, the absorption peak near 3420 cm^−1^ in all fermented samples shifted to a lower wavenumber, suggesting fermentation enhanced the number and strength of hydrogen bonds between starch molecules. This is consistent with results reported by Zhao et al. [33]. This may be due to fermentation producing short-chain starch molecules. These molecules possess strong intermolecular forces, promoting aggregation and allowing more hydroxyl groups to participate in hydrogen bond formation [15]. Furthermore, as the level of mixed culture increased, the intensity of the peak near 1538 cm^−1^ first increased and then decreased, suggesting the starch-protein complex content followed a similar trend. This may be because fermentation helps soften the bran, thereby reducing its hindrance to starch and protein, and promoting the formation of starch-protein complexes. However, microorganisms can metabolize proteins, potentially lowering protein content and subsequently reducing the starch protein complex [34].

The amide I band (1600~1700 cm^−1^) is commonly used to analyze protein secondary structure. The α-helix is an ordered structure stabilized by hydrogen bonds and contributes positively to dough elasticity and firmness. The β-sheet is a lamellar structure that requires extensive hydrogen bonding, also increasing dough springiness and hardness [35]. The β-turn and random coil are disordered structures with weak stability.

As shown in Table 2, compared to Y0-L0, Y1.0-L0.05 showed a significant reduction in the β-sheet content, and a notable increase in α-helix and β-turn content (*p* < 0.05), aligning with the results reported by Ge et al. [17]. The decrease in β-sheets indicates that fermentation increases protein flexibility, improves dough extensibility, and helps prevent noodle breakage by softening the bran and reducing fiber rigidity [36]. This is consistent with the reduced hardness observed in the texture analysis presented in Table 1. The increase in α-helix enhances the overall structural order and improves the elasticity of the noodle product, while an increase in β-turn content can improve dough cohesiveness and texture [36]. Therefore, fermentation with an appropriate amount of mixed culture shifts the protein secondary structure from β-sheets and random coils to α-helices and β-turns. This mitigates the structural hardening caused by bran and fiber and enhances the flexibility of the protein chains. This finding is consistent with research on the effect of yeast on the secondary structure of porous noodles [37].

### 3.6. Effect of Mixed Culture Addition on Crystallization Characteristics of Whole-Wheat Non-Fried Instant Noodles

Figure 3B displays the XRD patterns of non-fried whole-wheat instant noodles treated with different dosages of mixed culture, while Table 2 shows their corresponding relative crystallinity values. The XRD patterns of all samples exhibited a distinct diffraction peak near 20.0°(2θ), which corresponds to the characteristic diffraction of V-type starch-lipid complexes [38]. This demonstrates that mixed culture fermentation failed to change the crystal pattern of starch.

As shown in Table 2, compared to Y0-L0, mixed culture fermentation markedly lowered the relative crystallinity of the noodles (*p* < 0.05). This phenomenon is mainly due to amylases and organic acids generated in the fermentation process. These metabolites are capable of hydrolyzing starch glycosidic bonds and destroying the double-helix crystalline configuration of amylopectin, thus weakening the structural order of starch crystalline regions [39]. Furthermore, fermentation promotes the formation of protein-amylose complexes, which can impede the internal aggregation of starch molecules and inhibit the reordering of amylopectin short chains, thereby indirectly suppressing the crystallization process [40].

It is noteworthy that the crystallinity of Y1.0-L0.05 (26.00%) was significantly lower than that of Y1.0-L0 (27.57%), indicating an additional reinforcing effect of LAB in the deconstruction of the starch crystalline structure. Compared with the single-yeast fermentation system, the lactic acid produced by LAB metabolism lowers the pH of the system. This protonates the oxygen atoms of glycosidic bonds and disrupts the inter- and intra-molecular hydrogen bond networks that maintain the double-helix structure, ultimately leading to the disintegration of the ordered structure in the crystalline regions [41]. Furthermore, LAB can hydrolyze loose amylopectin branches out of the crystalline regions via enzymatic action, leading to a reduction in the crystal structure [42].

### 3.7. Effect of Mixed Culture Addition on Flavor of Non-Fried Whole-Wheat Instant Noodles

GC-MS analysis identified a total of 117 volatile compounds in non-fried whole-wheat instant noodles supplemented with different additions of mixed culture, as summarized in Table S2. These comprised 15 aldehydes, 30 alcohols, 26 esters, 25 hydrocarbons, five ketones, and 16 other compounds. The main volatile compounds detected in non-fried whole-wheat instant noodles fermented with varying amounts of mixed culture are presented in Figures S1–S5. The profiles of the identified volatile compounds remained consistent across different treatments, whereas their relative contents varied.

As shown in Figure 3C,D, mixed fermentation with yeast and LAB changed the variety and concentration of volatile compounds. Compared with Y0-L0, the number of volatile compound types in Y1.0-L0 decreased from 49 to 41, and the total content of volatile compounds dropped from 534.08 μg/kg to 303.73 μg/kg. In contrast, both the variety and total content of volatile compounds in Y1.0-L0.01 increased to 63 types and 440.86 μg/kg, respectively, compared to Y1.0-L0. In particular, the formation of esters, aldehydes, and alcohols was promoted, which compensated for the flavor deficiencies of single-yeast fermentation and masked the bitter taste caused by bran in whole-wheat flour. Consequently, characteristic flavors such as fruity and mellow notes became more balanced and abundant. This phenomenon was probably attributed to the organic acids produced by LAB, which reduced the system pH and activated cereal proteases. The activated proteases intensified protein hydrolysis in whole-wheat noodles and thereby promoted the production of amino acids [8]. Moreover, the results indicated that yeast exerted a minor direct effect on flavor, while LAB played a dominant role, which was consistent with the findings of Zhang et al. [12]. However, when the addition of LAB was further increased to 0.05% and 0.1%, the total number of flavor compounds decreased to 34 and 41, respectively, while the content of volatile compounds declined to 252.42 μg/kg and 273.63 μg/kg (a reduction of approximately 40%). This suggests that an excessively high proportion of mixed strains not only failed to improve the flavor but may instead inhibit the generation of flavor compounds. The underlying reason might be that over-acidification restrains general metabolism, thereby limiting metabolic routes and decreasing the abundance of volatile compounds [20].

Aldehydes are typically generated through lipid oxidation in flour, and high concentrations of aldehydes can result in off-flavors [43,44]. As indicated in Table S2, the variety of aldehydes increased after fermentation, mainly involving 2-heptenal, (2E,4E)-2,4-nonadienal, and (E)-2-hexadecenal. However, the aldehyde content decreased after fermentation (Figure 3D). In addition, although the alcohol content in the five noodle samples was relatively low, the diversity of alcohols increased with the addition of mixed strains, indicating that the mixed cultures play a crucial role in improving the overall flavor profile and shaping the characteristic flavor of the noodles. Notably, (2,2,6-trimethylbicyclo[4.1.0]hept-1-yl) methanol was detected only in samples produced by mixed fermentation with LAB and yeast, identifying it as a characteristic alcohol derived from this mixed fermentation process.

**Figure 4:**
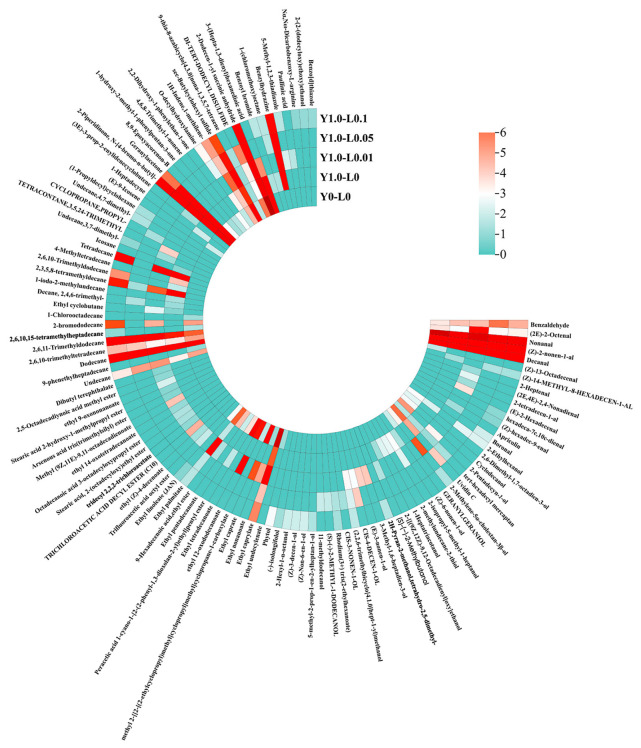
Heat map of volatiles clustering. Samples tested were of the control group (Y0-L0), 1.0% yeast with no lactic acid bacteria (LAB) (Y1.0-L0), 1.0% yeast with 0.01% LAB (Y1.0-L0.01), 1.0% yeast with 0.05% LAB (Y1.0-L0.05), and 1.0% yeast with 0.1% LAB (Y1.0-L0.1).

As shown in Figure 4, ethyl palmitate and ethyl caprylate were found in all five noodle groups. Ethyl caprylate, present in concentrations ranging from 4.34 μg/kg to 74.95 μg/kg (Table S2), imparts a brandy-like aroma [18] and contributes positively to noodle flavor. After fermentation, the variety of esters increased, notably with the addition of compounds such as ethyl (Z)-4-decenoate and ethyl 9-oxononanoate (Figure 4). These esters have low sensory thresholds and pleasant fruity aromas [45], enriching the overall flavor complexity of instant noodles.

Hydrocarbon content, consisting of long-chain alkanes like dodecane and tetradecane, was relatively high. Alkanes generally have high sensory thresholds and contribute little direct aroma [46]. However, they may indirectly influence the overall aroma profile through the synergistic effect between compounds [47]. As shown in Figure 4, the slight increase in hydrocarbon content after fermentation may contribute to flavor enrichment.

Ketones, which have low odor thresholds, significantly impact the aroma of whole-wheat instant noodles [48]. Among them, geranyl acetone contributes sweet, magnolia-like, and fresh fragrances [49,50], playing an important role in noodles’ flavor profile (Figure 4).

Collectively, co-fermentation with yeast and LAB promotes the formation of flavor compounds, with LAB playing a predominant role. These compounds primarily included aldehydes (e.g., 2-heptenal, (2E,4E)-2,4-nonadienal), alcohols (e.g., (2,2,6-trimethylbicyclo[4.1.0]hept-1-yl) methanol, cis-4-decen-1-ol), and esters (e.g., ethyl (Z)-4-decenoate, 9-oxononanoate), which can effectively mask the bran off-flavor of the whole-wheat system, endowing non-fried whole-wheat instant noodles with a unique and pleasant flavor.

### 3.8. Effects of Mixed Culture Addition on Digestive Properties of Non-Fried Whole-Wheat Instant Noodles

**Figure 5:**
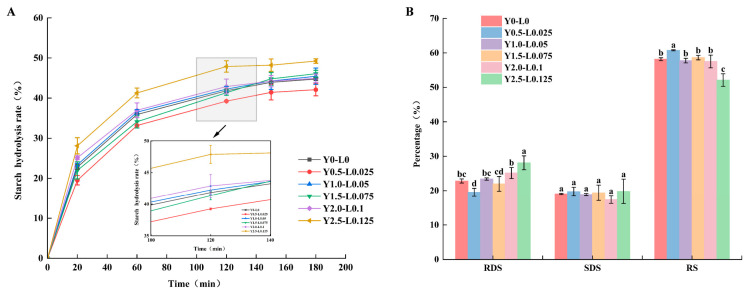
Effect of mixed culture addition on starch digestion properties of non-fried whole-wheat instant noodles. (**A**) Starch hydrolysis rate, (**B**) starch percentage. Bars with different letters indicate statistically significant differences between each other (*p* < 0.05). Samples tested were of the control group (Y0-L0), 0.5% yeast with 0.025% lactic acid bacteria (LAB) (Y0.5-L0.025), 1.0% yeast with 0.05% LAB (Y1.0-L0.05), 1.5% yeast with 0.075% LAB (Y1.5-L0.075), 2.0% yeast with 0.1% LAB (Y2.0-L0.1), and 2.5% yeast with 0.125% LAB (Y2.5-L0.125).

The effect of mixed culture addition on the starch digestibility is shown in Figure 5A. All samples exhibited a highly consistent trend in the starch hydrolysis over time. Specifically, the hydrolysis rate peaked within the first 20 min, representing the most rapid digestion phase. From 20 to 120 min, the hydrolysis rate slowed significantly compared to the initial stage. After 120 min, the hydrolysis curve gradually plateaued, indicating the completion of the starch digestion process, which is consistent with the observations of Chang et al. [51].

As shown in Figure 5A, the Y2.5-L0.125 exhibited the highest digestion rate, while the Y0.5-L0.025 group showed the lowest. No significant differences were observed among Y2.0-L0.1, Y1.0-L0.05, Y0-L0, and Y1.5-L0.075. The high digestibility of Y2.5-L0.125 is closely associated with its loose and porous microstructure (Figure 2). Yeast fermentation increased the number and size of pores, thereby enhancing the accessibility of digestive enzymes to the starch [51]. Furthermore, FTIR and XRD analyses indicated that excessive fermentation might reduce the short-range order and relative crystallinity of starch, further promoting enzymatic efficiency and thereby facilitating starch digestion. In contrast, the dense network structure of Y0.5-L0.025 (Figure 2) limited the water absorption and swelling of starch granules, hindering the contact between starch and digestive enzymes, and thus resulting in the lowest hydrolysis rate [52].

Furthermore, as shown in Figure 5B, the trend in RDS content aligned with the starch hydrolysis rate across different mixed culture additions, while RS content showed an inverse relationship. SDS content remained relatively stable at all addition levels, ranging from approximately 18–20%.

The eGI values and starch digestion kinetics are presented in Table 3. The high *R*^2^ values (>0.98) indicate a good fitting effect of the kinetic model and high reliability of starch digestion data. According to Chang et al. [51], higher $C_{\infty }$ and *k* values correspond to a faster in vitro starch digestion rate. Compared to Y0-L0, Y0.5-L0.025 had the lowest $C_{\infty }$ (41.46%) value, while Y2.5-L0.125 gained the highest $C_{\infty }$ (48.13%) and *k* (0.04 min^−1^) values. This trend cohered with the eGI values, suggesting that an appropriate addition of mixed culture may increase RS content, retard starch hydrolysis, and slow glucose release, thereby having the lowest eGI. Conversely, excessive addition may reduce RS content, while increasing RDS content, which accelerates starch digestibility and promotes rapid glucose release, leading to an elevated eGI in non-fried instant whole-wheat noodles. This result corroborates the variation pattern of starch crystallinity revealed by XRD (Table 2). As the addition level of mixed cultures increased, starch relative crystallinity gradually decreased on the whole. Y2.5-L0.125 exhibited the lowest crystallinity (25.53%) and the loosest starch structure, which promoted the accessibility of digestive enzymes to starch molecules. In contrast, Y0.5-L0.025 maintained a higher level of crystallinity and formed a dense gluten network, which hindered the enzymatic hydrolysis of starch.

According to the standard classification, foods with eGI ≤ 55 are considered low-eGI. As shown in Table 3, the eGI values of all non-fried whole-wheat instant noodles were approximately 40, categorizing them as low-eGI foods. This is consistent with the findings that the predominant starch component in these noodles is RS (Figure 5B). Therefore, non-fried whole-wheat instant noodles possess potential as a supplementary food for individuals with diabetes.

## 4. Conclusions

This study investigated the effects of mixed culture fermentation on the edible and digestive properties of non-fried whole-wheat instant noodles. The results determined that the optimal addition level of mixed bacteria was 1% yeast and 0.05% LAB. The study further revealed that yeast predominantly influenced the rehydration and textural characteristics, while LAB were primarily responsible for flavor enhancement.

Fermentation with a moderate amount of mixed culture repaired the structural defects of the whole-wheat system, shortened the rehydration time, and endowed the noodles with a uniform, porous microstructure. It also increased the variety of volatile compounds, enriched the aroma, and reduced the starch digestion rate, ultimately leading to a lower eGI. Conversely, excessive fermentation damaged the integrity of the gluten network and exacerbated the loosening of the whole-wheat structure, which increased the accessibility of starch to digestive enzymes, thus leading to an upward trend in the eGI values.

Co-fermentation with yeast and LAB offers a novel and feasible strategy for producing high-quality non-fried whole-wheat instant noodles. This approach effectively addresses the defects of bran interference and deteriorated fiber texture in whole-wheat, enabling the development of palatable, low-eGI staple foods for health-conscious and glycemic-controlled populations. However, this study was limited to investigating the effects of these two common microorganisms. Future research could explore other fermentation strains to further improve noodle quality. Additionally, combining whole-wheat flour with beans, cereals, and potatoes could be pursued to optimize the nutritional profile of composite grain formulations.

## Figures and Tables

**Table 1:** Effect of mixed culture addition on textural characteristics of non-fried whole-wheat instant noodles.

Samples	Hardness (gf)	Adhesiveness (gf∙s)	Springiness	Chewiness (gf)	Shear Force (gf)	Toughness (gf∙s)	Tensile Strength (gf)	Elongation at Break (%)
Y0-L0	2393.31 ± 21.55 ^a^	13.04 ± 1.53 ^a^	0.85 ± 0.01 ^a^	1568.95 ± 73.16 ^a^	218.37 ± 6.47 ^a^	123.78 ± 4.61 ^a^	17.84 ± 0.73 ^a^	1.50 ± 0.01 ^a^
Y0.5-L0.025	1940.94 ± 44.30 ^b^	11.54 ± 2.11 ^ab^	0.82 ± 0.02 ^ab^	1255.25 ± 37.85 ^b^	199.66 ± 3.48 ^b^	113.17 ± 2.30 ^bc^	14.76 ± 0.70 ^b^	1.50 ± 0.01 ^a^
Y1.0-L0.05	1858.01 ± 17.22 ^b^	9.27 ± 1.20 ^bc^	0.82 ± 0.02 ^ab^	1205.31 ± 52.00 ^b^	181.71 ± 3.67 ^d^	109.55 ± 1.72 ^c^	13.29 ± 1.03 ^c^	1.50 ± 0.00 ^a^
Y1.5-L0.075	1416.29 ± 35.46 ^cd^	9.23 ± 1.48 ^bc^	0.80 ± 0.03 ^b^	887.25 ± 5.18 ^c^	151.86 ± 2.20 ^ef^	84.78 ± 2.46 ^d^	11.59 ± 0.19 ^d^	1.45 ± 0.06 ^a^
Y2.0-L0.1	1502.58 ± 95.06 ^c^	9.28 ± 2.43 ^bc^	0.79 ± 0.01 ^b^	932.47 ± 62.31 ^c^	157.30 ± 0.92 ^e^	85.88 ± 3.30 ^d^	13.35 ± 0.09 ^c^	1.45 ± 0.08 ^a^
Y2.5-L0.125	1338.97 ± 40.42 ^d^	8.22 ± 1.19 ^c^	0.75 ± 0.04 ^c^	768.07 ± 46.57 ^d^	149.12 ± 4.63 ^f^	86.85 ± 6.84 ^d^	10.67 ± 0.27 ^d^	1.41 ± 0.06 ^a^
Y1.0-L0	1851.61 ± 0.69 ^b^	9.20 ± 0.17 ^bc^	0.81 ± 0.01 ^ab^	1195.75 ± 0.96 ^b^	188.73 ± 1.61 ^c^	117.85 ± 5.37 ^ab^	13.68 ± 1.30 ^bc^	1.46 ± 0.03 ^a^

Note: Values with different superscripts in the same row are significantly different at *p* < 0.05. The results are expressed as mean ± SD (*n* = 3). Samples tested were of the control group (Y0-L0), 0.5% yeast with 0.025% lactic acid bacteria (LAB) (Y0.5-L0.025), 1.0% yeast with 0.05% LAB (Y1.0-L0.05), 1.5% yeast with 0.075% LAB (Y1.5-L0.075), 2.0% yeast with 0.1% LAB (Y2.0-L0.1), and 2.5% yeast with 0.125% LAB (Y2.5-L0.125).

**Table 2:** Effect of mixed culture addition on secondary protein structure and crystallinity of non-fried whole-wheat instant noodles.

Samples	β-Sheet (%)	Random Coil (%)	α-Helix (%)	β-Turn (%)	Crystallinity (%)
Y0-L0	48.58 ± 0.16 ^a^	17.74 ± 0.04 ^a^	17.43 ± 0.09 ^b^	33.99 ± 0.25 ^b^	29.23 ± 0.32 ^a^
Y0.5-L0.025	48.60 ± 0.86 ^a^	17.56 ± 0.14 ^a^	17.40 ± 0.08 ^b^	34.00 ± 0.78 ^b^	26.90 ± 0.47 ^c^
Y1.0-L0.05	46.83 ± 0.00 ^b^	17.54 ± 0.08 ^ab^	17.88 ± 0.02 ^a^	35.30 ± 0.01 ^a^	26.00 ± 0.18 ^d^
Y2.5-L0.125	48.71 ± 0.23 ^a^	17.18 ± 0.40 ^b^	17.19 ± 0.44 ^b^	34.10 ± 0.21 ^b^	25.53 ± 0.45 ^d^
Y1.0-L0	47.94 ± 0.23 ^a^	17.58 ± 0.09 ^a^	17.56 ± 0.10 ^ab^	34.50 ± 0.14 ^b^	27.57 ± 0.28 ^b^

Note: Values with different superscripts in the same row are significantly different at *p* < 0.05. The results are expressed as mean ± SD (*n* = 3). Samples tested were of the control group (Y0-L0), 0.5% yeast with 0.025% lactic acid bacteria (LAB) (Y0.5-L0.025), 1.0% yeast with 0.05% LAB (Y1.0-L0.05), 2.5% yeast with 0.125% LAB (Y2.5-L0.125), and 1.0% yeast with no LAB (Y1.0-L0).

**Table 3:** Effect of mixed culture addition on starch digestion kinetic parameters and eGI values of non-fried whole-wheat instant noodles.

Samples	*C*_∞_	*k* (min^−1^)	*R* ^2^	eGI
Y0-L0	43.63	0.03	0.99291	39.41
Y0.5-L0.025	41.46	0.03	0.99593	37.12
Y1.0-L0.05	44.06	0.03	0.9922	39.83
Y1.5-L0.075	44.59	0.03	0.98451	38.97
Y2.0-L0.1	43.82	0.04	0.99107	40.33
Y2.5-L0.125	48.13	0.04	0.99322	43.81

Note: Samples tested were of control group (Y0-L0), 0.5% yeast with 0.025% lactic acid bacteria (LAB) (Y0.5-L0.025), 1.0% yeast with 0.05% LAB (Y1.0-L0.05), 1.5% yeast with 0.075% LAB (Y1.5-L0.075), 2.0% yeast with 0.1% LAB (Y2.0-L0.1), and 2.5% yeast with 0.125% LAB (Y2.5-L0.125).

## Data Availability

The original contributions presented in the study are included in the article; further inquiries can be directed to the corresponding authors.

## Supplementary Materials

The following supporting information can be downloaded at https://www.mdpi.com/article/10.3390/foods15132265/s1, Table S1. Sensory evaluation table for non-fried whole-wheat instant noodles. Table S2. Volatile compounds and their relative content in non-fried whole-wheat instant noodles. Figure S1: Gas chromatogram of Y0-L0 (non-fried whole wheat instant noodles without mixed-culture fermentation). Figure S2: Gas chromatogram of Y1.0-L0 (non-fried whole-wheat instant noodles fermented exclusively with 1.0% yeast). Figure S3: Gas chromatogram of Y1.0-L0.01 (non-fried whole-wheat instant noodles fermented with 1.0% yeast and 0.01% lactic acid bacteria). Figure S4: Gas chromatogram of Y1.0-L0.05 (non-fried whole-wheat instant noodles fermented with 1.0% yeast and 0.05% lactic acid bacteria). Figure S5: Gas chromatogram of Y1.0-L0.1 (non-fried whole-wheat instant noodles fermented with 1.0% yeast and 0.1% lactic acid bacteria).

## Abbreviations

RDS, rapidly digestible starch; SDS, slowly digestible starch; RS, resistant starch; eGI, expected glycemic index; SEM, scanning electron microscope; FTIR, Fourier transform infrared spectra; XRD, X-ray diffraction; GC-MS, Gas Chromatography–Mass Spectrometry.
