# Supplementary material for: Effects of Mixed Culture Fermentation on Quality of Non-Fried Whole-Wheat Instant Noodles
Source: Foods. 2026 Jun 24;15(13):2265. doi: 10.3390/foods15132265 (PMC13360998; doi:10.3390/foods15132265)
Supplement: Supplementary file 1 [file foods-15-02265-s001.zip › foods-4300565-supplementary.pdf]

Supplementary materials

Table S1 Sensory evaluation table for non-fried whole wheat instant noodles.

Indicators	Score	Grading Criteria
Color	10	• Light Buff with Wheat Bran Undertones: 8-10<br>• Burnish of general: 5–7<br>• Brightness difference: 1-4
Appearance	10	• Tight and smooth: 8-10<br>• Slight defect in appearance: 5-7<br>• Tough and shape change: 1-4
Hardness	10	• Suitable hardness and softness: 8-10<br>• Moderate: 5-7<br>• Improper hardness and softness: 1-4
Stickiness	15	• Tasting good when chewing: 13-15<br>• Sticky: 10-12<br>• Clammy: 1-9
Smoothness	15	• Smooth: 13-15<br>• Moderate: 10-12<br>• Poor smoothness: 1-9
Taste	20	•Whole wheat toasty nutty sweetness: 15-20<br>•Moderate: 10-14<br>•Floury-raw with lingering cereal bitterness: 1-9
Flavor	20	• Rich fermentation aromas: 15-20<br>• Moderate: 10-14<br>• Off-flavor: 1-9

Table S2 Volatile compounds and their relative content in non-fried whole wheat instant noodles.

Categories	Volatile compounds	Relative content of flavor compounds（ug/kg）				
		Y0-L0	Y1.0-L0	Y1.0-L0.01	Y1.0-L0.05	Y1.0-L0.1
Aldehydes	Benzaldehyde	3.55±0.01^{d}	4.39±0.02^{c}	4.40±0.01^{c}	6.14±0.01^{a}	4.78±0.01^{b}
	(2E)-2-Octenal	3.62±0.01^{b}	2.84±0.02^{e}	8.35±0.01^{a}	3.09±0.01^{d}	3.30±0.01^{c}
	Nonanal	72.83±0.01^{a}	43.52±0.02^{e}	60.66±0.02^{b}	44.75±0.02^{c}	44.32±0.03^{d}
	(Z)-2-nonen-1-al	27.86±0.02^{a}	15.17±0.02^{d}	23.55±0.01^{b}	12.86±0.01^{e}	16.77±0.02^{c}
	Decanal	25.75±0.02^{a}	12.27±0.02^{c}	16.31±0.02^{b}	10.45±0.02^{e}	12.18±0.01^{d}
	(Z)-13-Octadecenal	1.79±0.01				
	(Z)-14-METHYL-8-HEXADECEN-1-AL		1.27±0.01^{c}		1.46±0.02^{a}	1.32±0.03^{b}
	2-Heptenal			1.10±0.02		
	(2E,4E)-2,4-Nonadienal			2.44±0.01		
	2-tetradecen-1-al			1.18±0.15		

Categories	Volatile compounds	Relative content of flavor compounds（ug/kg）				
		Y0-L0	Y1.0-L0	Y1.0-L0.01	Y1.0-L0.05	Y1.0-L0.1
Aldehydes	(E)-2-Hexadecenal			3.66±0.12		
	hexadeca-7c,10c-dienal					0.85±0.01
	(Z)-hexadec-9-enal					0.75±0.02
	Apricolin			1.20±0.01		
	Boronal					2.09±0.01
Alcohols	2-Ethylhexanol	3.04±0.04^{a}	2.28±0.00^{d}		2.51±0.02^{b}	2.34±0.02^{c}
	2,6-Dimethyl-1,7-octadien-3-ol	2.43±0.02				
	Cyclodecanol	2.78±0.02				
	2-Pentadecyn-1-ol	5.38±0.02^{a}		3.22±0.02^{b}		
	tert-hexadecyl mercaptan	1.59±0.03^{a}	1.39±0.03^{b}	1.11±0.02^{d}	1.32±0.03^{c}	
Alcohols	Uvidin C	6.32±0.02^{a}	4.91±0.03^{b}		4.15±0.03^{c}	

Categories	Volatile compounds	Relative content of flavor compounds（ug/kg）				
		Y0-L0	Y1.0-L0	Y1.0-L0.01	Y1.0-L0.05	Y1.0-L0.1
	2-Methylene-5α-cholestan-3β-ol	3.25±0.01[b]		6.83±0.02[a]		1.45±0.03[c]
	GERANYLGERANIOL	2.03±0.02				
	(Z)-6-nonen-1-ol		1.42±0.02			
	2-isopropyl-5-methyl-1-heptanol		2.92±0.02[a]	2.44±0.02[b]		
	2-methylundecane-2-thiol		2.55±0.03			
	1-Heptatriacotanol		2.39±0.03			
	2-[[(9Z,12Z)-9,12-Octadecadienyl]oxy]ethanol		1.22±0.02			
	(S)-(−)-2-Methylbutanol			1.49±0.02		
	2H-Pyran-2-methanol,tetrahydro-2,5-dimethyl-			1.72±0.03		
Alcohols	3-Methyl-1,6-heptadien-3-ol			4.22±0.02		
	(E)-3-nonen-1-ol			4.03±0.03		

Categories	Volatile compounds	Relative content of flavor compounds（ug/kg）				
		Y0-L0	Y1.0-L0	Y1.0-L0.01	Y1.0-L0.05	Y1.0-L0.1
	CIS-4-DECEN-1-OL			2.90±0.02^{b}	4.14±0.03^{a}	
	(2,2,6-trimethylbicyclo[4.1.0]hept-1-yl)methanol			3.13±0.02^{a}	2.27±0.03^{b}	1.74±0.03^{c}
	CIS-3-NONEN-1-OL				1.26±0.02	
	Rhodium(3+) tris(2-ethylhexanoate)				1.46±0.02^{a}	1.28±0.00^{b}
	(S)-(-)-2-METHYL-1-DODECANOL				1.86±0.02	
	11-methyldodecanol				1.91±0.03	
	2H-Pyran-2-methanol,tetrahydro-2,5-dimethyl-					1.60±0.01
	5-methyl-2-prop-1-en-2-ylheptan-1-ol					1.43±0.02
	(Z)-Non-6-en-1-ol					2.09±0.03
Alcohols	(Z)-3-decen-1-ol					2.03±0.03
	2-Hexyl-1-n-octanol					2.02±0.03

Categories	Volatile compounds	Relative content of flavor compounds（ug/kg）				
		Y0-L0	Y1.0-L0	Y1.0-L0.01	Y1.0-L0.05	Y1.0-L0.1
	(-)-isolongifolol					1.18±0.02
	Phytol					2.81±0.03
Esters	Ethyl undecylenate	1.17±0.03				
	Ethyl caprylate	74.95±0.05^{a}	6.69±0.03^{c}	25.82±0.02^{b}	4.34±0.03^{e}	5.74±0.04^{d}
	Ethyl nonanoate	1.13±0.03^{b}		4.98±0.03^{a}		
	Ethyl caprate	16.79±0.04^{a}		6.87±0.03^{b}		
	methyl 2-[[2-[(2-ethylcyclopropyl)methyl]cyclopropyl]methyl]cyclopropane-1-carboxylate	1.75±0.05				
	ethyl 12-oxododecanoate	8.13±0.03^{b}		12.24±0.04^{a}		
Esters	Ethyl tetradecanoate	13.06±0.05				
	Peracetic acid 1-cyano-1-[2-(2-phenyl-1,3-dioxolan-2-yl)ethyl]pentyl ester	3.70±0.02^{a}		1.77±0.03^{b}		

Categories	Volatile compounds	Relative content of flavor compounds（ug/kg）				
		Y0-L0	Y1.0-L0	Y1.0-L0.01	Y1.0-L0.05	Y1.0-L0.1
	Ethyl pentadecanoate	2.03±0.04				
	9-Hexadecenoic acid,ethyl ester	4.42±0.02[a]		1.45±0.03[b]		
	Ethyl palmitate	6.58±0.02[b]	1.78±0.03[c]	8.64±0.04[a]	1.71±0.03[d]	1.59±0.03[e]
	Ethyl linoleate (JAN)	2.83±0.04[a]				0.77±0.01[b]
	Trifluoroacetic acid octyl ester			1.88±0.02		
	ethyl (Z)-4-decenoate			1.28±0.03		
	TRICHLOROACETIC ACID DECYL ESTER (C10)			4.74±0.04		
	tridecyl 2,2,2-trichloroacetate			1.23±0.03		
Esters	Stearic acid, 2-(octadecyloxy)ethyl ester			1.35±0.05		
	Octadecanoic acid 3-octadecyloxypropyl ester			1.41±0.02		
	ethyl 14-oxotetradecanoate			1.02±0.03		

Categories	Volatile compounds	Relative content of flavor compounds（ug/kg）				
		Y0-L0	Y1.0-L0	Y1.0-L0.01	Y1.0-L0.05	Y1.0-L0.1
	Methyl (9Z,11E)-9,11-octadecadienoate			4.33±0.03		
	Arsenous acid tris(trimethylsilyl) ester			0.74±0.04		
	Stearic acid 2-hydroxy-1-methylpropyl ester			0.75±0.01		
	tridecyl 2,2,2-trichloroacetate			0.89±0.03		
	ethyl 9-oxononanoate			2.21±0.03		
	2,5-Octadecadiynoic acid methyl ester			0.90±0.03		
	Dibutyl terephthalate			0.73±0.03		
Hydrocarbons	Undecane	5.04±0.04^{c}	3.34±0.04^{e}	5.41±0.04^{a}	5.16±0.04^{b}	3.47±0.05^{d}
Hydrocarbons	9-phenethylheptadecane	1.04±0.05				
	Dodecane	11.81±0.03^{e}	14.80±0.04^{d}	15.70±0.03^{c}	17.03±0.03^{b}	17.35±0.02^{a}
	2,6,10-trimethyltetradecane	5.07±0.06^{a}	3.54±0.04^{d}	3.19±0.05^{e}	4.22±0.03^{c}	4.55±0.05^{b}

Categories	Volatile compounds	Relative content of flavor compounds（ug/kg）				
		Y0-L0	Y1.0-L0	Y1.0-L0.01	Y1.0-L0.05	Y1.0-L0.1
	2,6,11-Trimethyldodecane	6.57±0.03^{e}	7.99±0.03^{d}	10.53±0.03^{b}	14.80±0.03^{a}	9.60±0.03^{c}
	2,6,10,15-tetramethylheptadecane	4.18±0.07^{a}				0.92±0.03^{b}
	2-bromododecane	5.17±0.03^{b}		4.81±0.04^{c}		7.03±0.06^{a}
	1-Chlorooctadecane	1.17±0.07				
	2,6,10,15-tetramethylheptadecane	1.41±0.02^{a}				0.89±0.08^{b}
	Ethyl cyclobutane		0.75±0.02^{b}	2.45±0.03^{a}		
	Decane, 2,4,6-trimethyl-		1.11±0.04^{b}		1.40±0.04^{a}	
	1-iodo-2-methylundecane		1.30±0.10			
Hydrocarbons	2,3,5,8-tetramethyldecane		10.71±0.04^{a}	6.68±0.05^{c}	1.23±0.04^{d}	7.97±0.03^{b}
	2,6,10-Trimethyldodecane		1.82±0.02^{b}			5.48±0.02^{a}
	4-Methyltetradecane		4.29±0.08			

Categories	Volatile compounds	Relative content of flavor compounds（ug/kg）				
		Y0-L0	Y1.0-L0	Y1.0-L0.01	Y1.0-L0.05	Y1.0-L0.1
	Tetradecane		12.47±0.03^{c}	14.19±0.03^{a}		13.21±0.02^{b}
	Icosane		0.76±0.10			
	Undecane,3,7-dimethyl-			1.17±0.03^{a}		0.88±0.03^{b}
	TETRACONTANE,3,5,24-TRIMETHYL			4.01±0.10		
	CYCLOPROPANE,PROPYL-				1.29±0.09	
	Undecane,4,7-dimethyl-					1.45±0.05
	(1-Propyldecyl)cyclohexane					1.27±0.08
	(E)-9-Icosene	2.07±0.01				
Hydrocarbons	1-Heptadecyne		2.00±0.10			
	(3E)-3-prop-2-enylidenecyclobutene			1.22±0.07		
Ketones	2-Piperidinone, N-[4-bromo-n-butyl]-	42.53±0.04^{a}	37.05±0.05^{c}	40.51±0.09^{b}	34.88±0.08^{d}	31.97±0.07^{e}

Categories	Volatile compounds	Relative content of flavor compounds（ug/kg）				
		Y0-L0	Y1.0-L0	Y1.0-L0.01	Y1.0-L0.05	Y1.0-L0.1
	Geranylacetone	17.61±0.06[a]	9.16±0.07[d]	11.28±0.07[c]	11.60±0.10[b]	6.15±0.05[e]
	8,9-Epoxyacorenon-B	2.78±0.08				
	1-hydroxy-2-methyl-1-phenylpentan-3-one	1.27±0.07				
	4,6,8-Trimethyl-1-nonene	1.63±0.07				
Others	2,2-Dihydroxy-1-phenylethan-1-one	2.84±0.05				
	O-decylhydroxylamine	1.77±0.08[d]	3.23±0.07[a]		2.49±0.09[c]	3.17±0.07[b]
	1H-Indene,1-methilene-	4.69±0.08[c]	4.18±0.06[e]	6.46±0.06[a]	4.35±0.05[d]	5.15±0.05[b]
	sec-Butylcyclohexyl sulfide	10.15±0.05[a]	7.61±0.01[d]	9.95±0.05[b]	8.16±0.07[c]	7.07±0.07[e]
Others	9-thia-8-azabicyclo[4.3.0]nona-1,3,5,7-tetraene	1.77±0.07[a]	1.15±0.05[c]	1.12±0.07[d]	1.32±0.07[b]	
	DI-TERT-DODECYL DISULFIDE	6.06±0.05[a]	3.03±0.06[d]	3.41±0.05[c]	5.78±0.01[b]	
	2-Dodecen-1-yl succinic anhydride	89.77±0.07[a]	44.55±0.05[b]	43.89±0.09[c]	17.90±0.10[d]	9.32±0.08[e]

Categories	Volatile compounds	Relative content of flavor compounds（ug/kg）				
		Y0-L0	Y1.0-L0	Y1.0-L0.01	Y1.0-L0.05	Y1.0-L0.1
	3-(Hepta-1,3-dienyl)hexanedioic acid	8.95±0.05^{a}	8.77±0.08^{b}	5.57±0.10^{c}		
	Benzoyl bromide		1.43±0.07^{b}			1.65±0.05^{a}
	1-(chloromethoxy)octane		1.41±0.06^{b}		1.61±0.06^{a}	1.61±0.06^{a}
	Benzylhydrazine		10.26±0.05^{c}	12.55±0.08^{b}	13.49±0.10^{a}	8.35±0.05^{d}
	5-Methyl-1,2,3-thiadiazole			2.24±0.10^{a}		1.61±0.10^{b}
	Paullinic acid			1.59±0.09		
	Nα,Nω-Dicarbobenzoxy-L-arginine					0.88±0.03
	2-(2-(dodecyloxy)ethoxy)ethanol					1.02±0.07
Others	Benzo[d]thiazole					1.00±0.01

Note: Values with different superscripts in the same row are significantly different at $p < 0.05$. The re-sults are expressed as mean ± SD (n = 3).

Figures

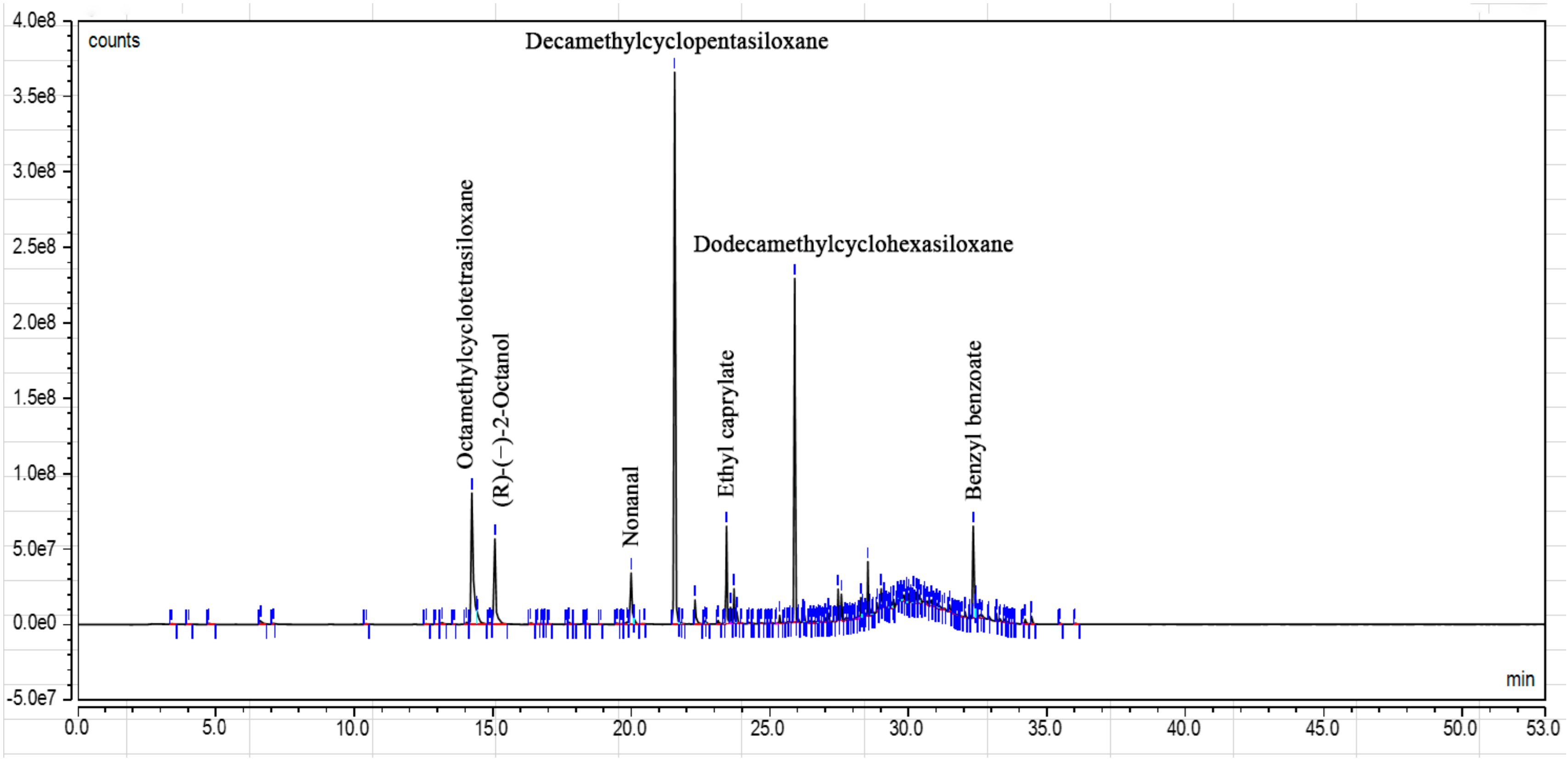

Figure S1 Gas chromatogram of Y0-L0 (non-fried whole wheat instant noodles without mixed-culture fermentation).

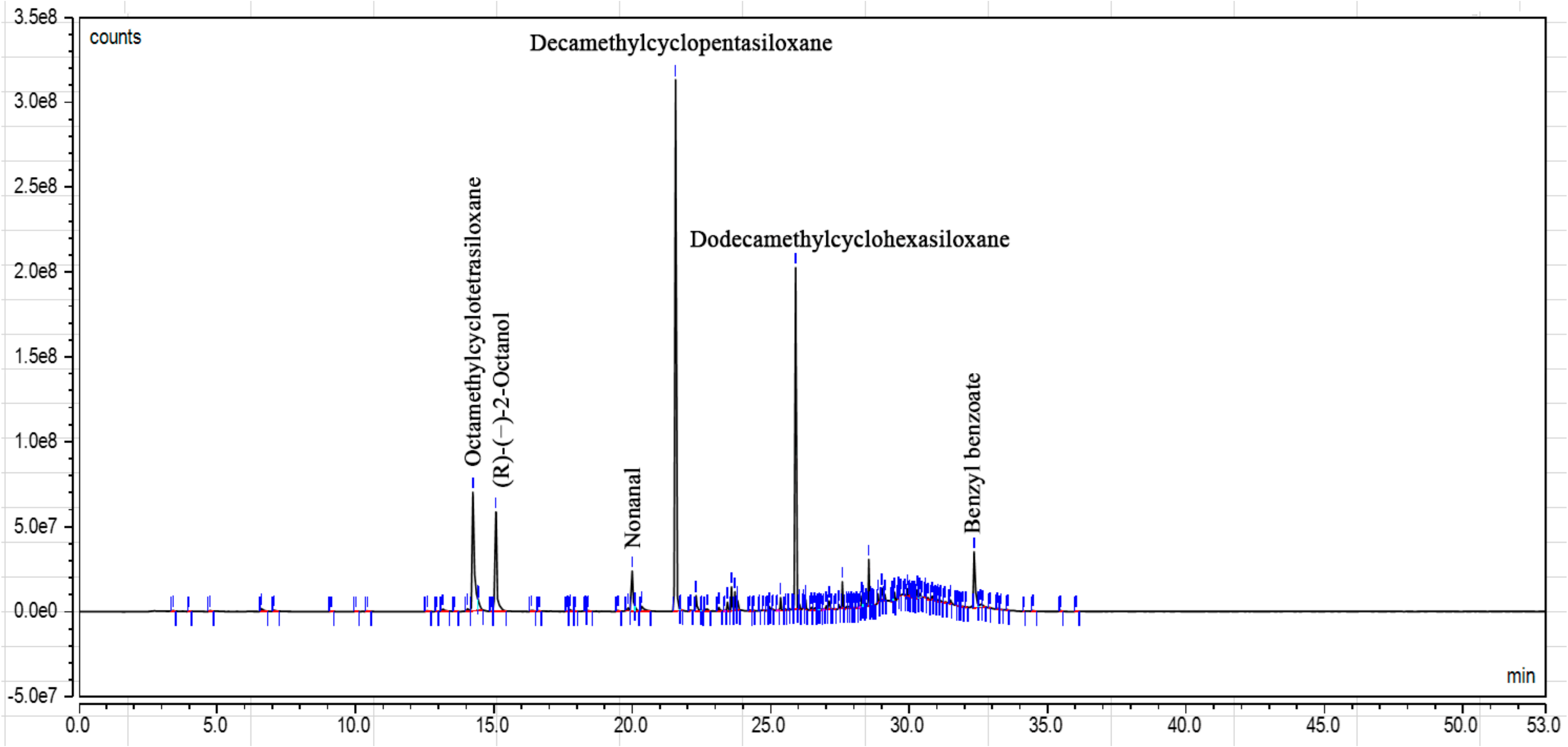

Figure S2 Gas chromatogram of Y1.0-L0 (non-fried whole wheat instant noodles fermented exclusively with 1.0% yeast).

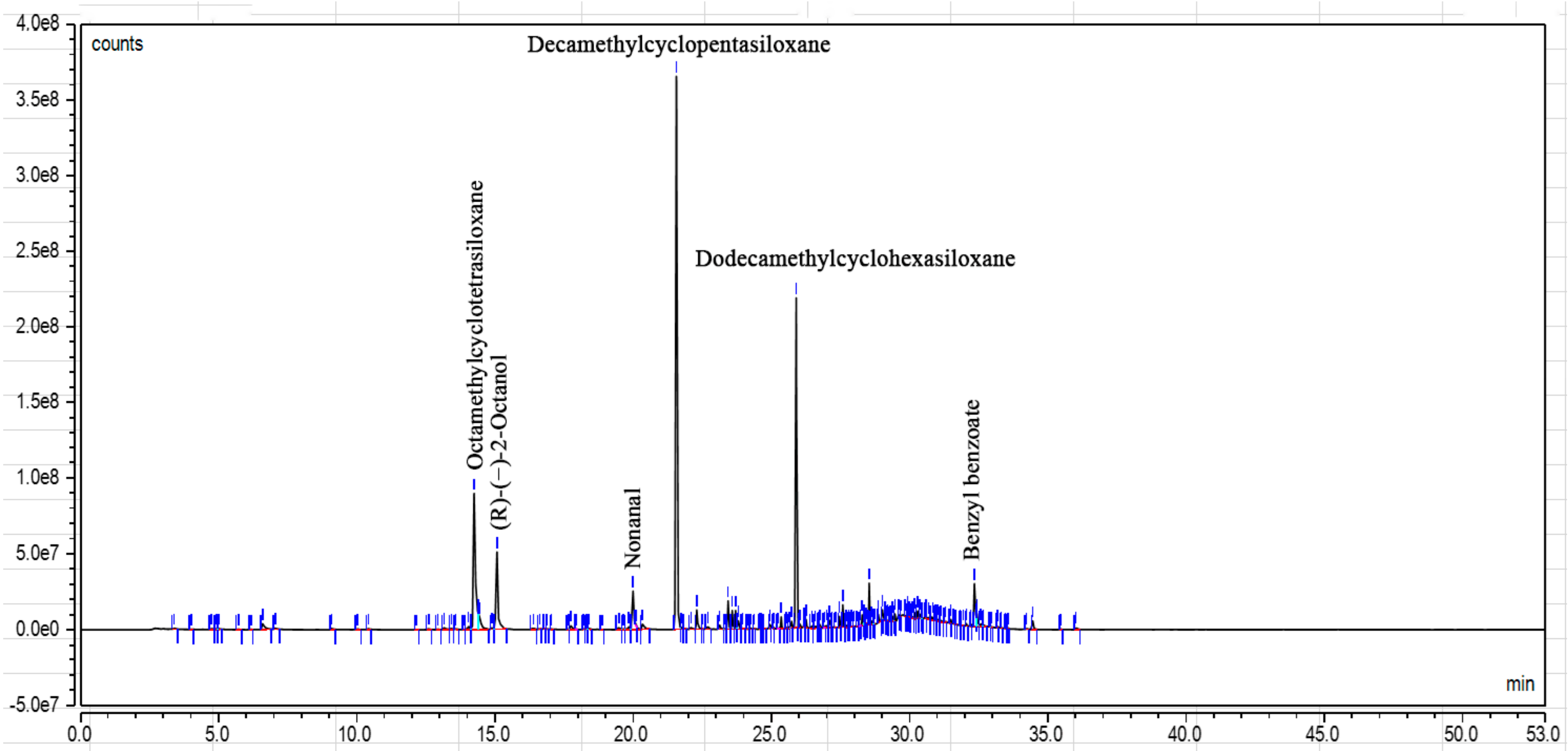

Figure S3 Gas chromatogram of Y1.0-L0.01 (non-fried whole wheat instant noodles fermented with 1.0% yeast and 0.01% lactic acid bacteria).

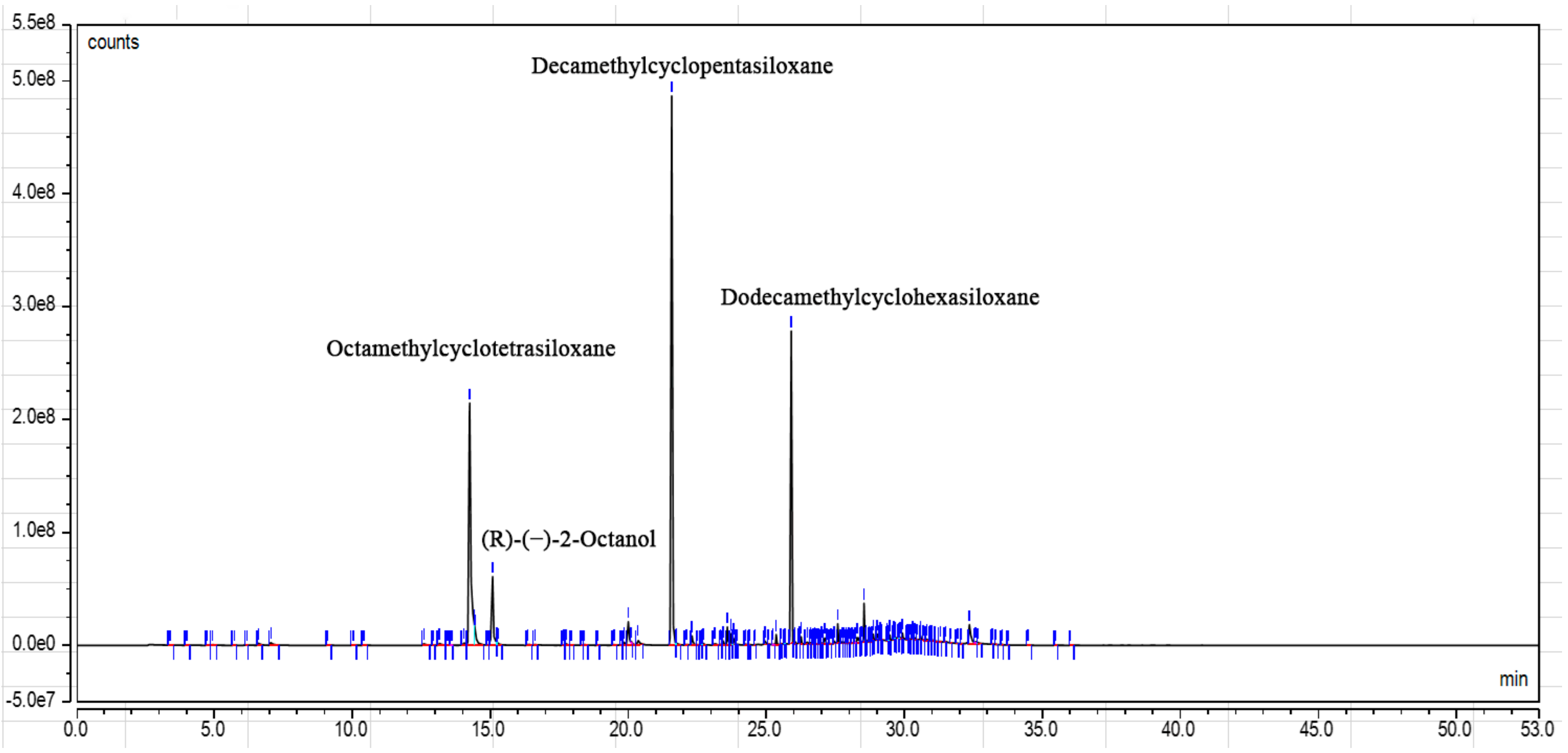

Figure S4 Gas chromatogram of Y1.0-L0.05 (non-fried whole wheat instant noodles fermented with 1.0% yeast and 0.05% lactic acid bacteria).

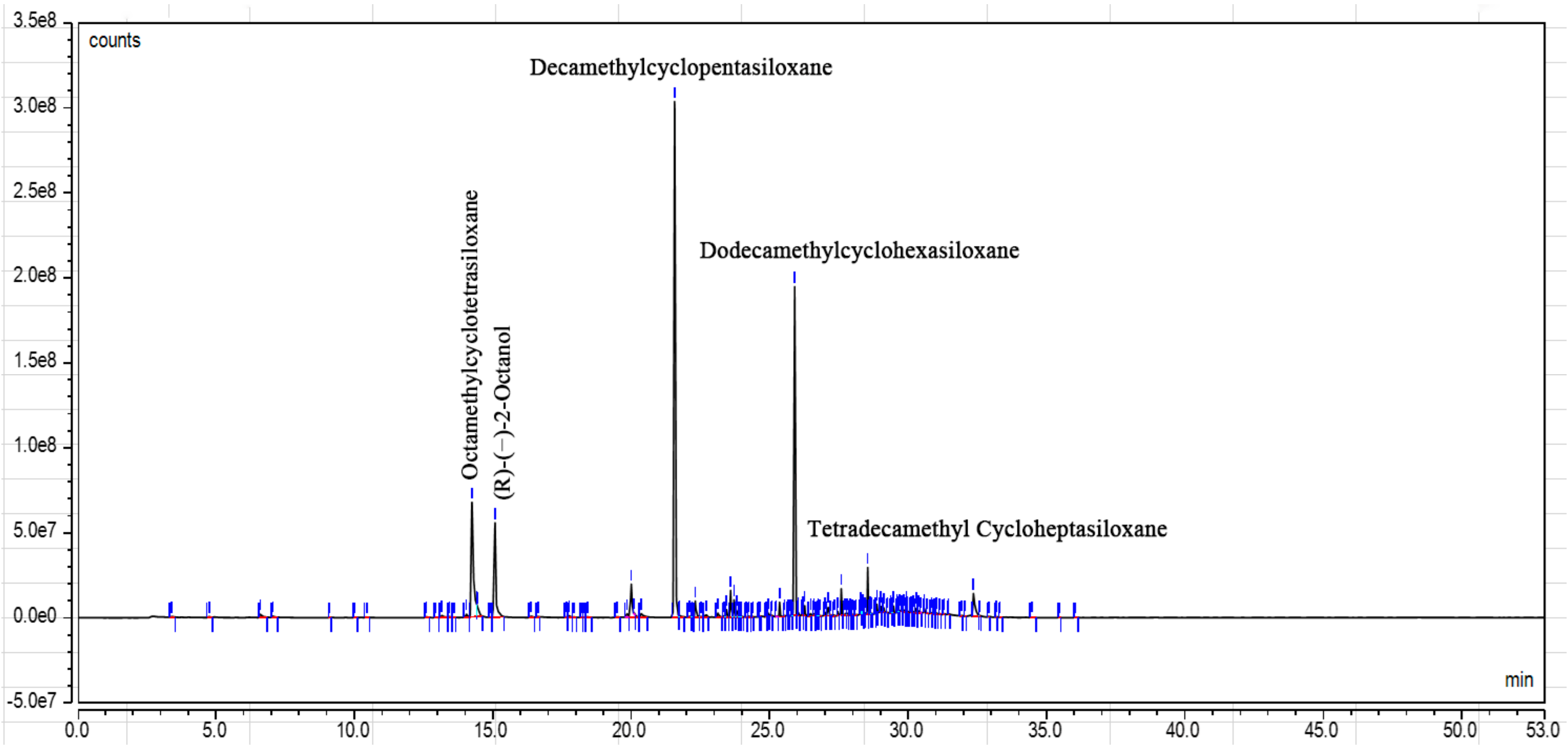

Figure S5 Gas chromatogram of Y1.0-L0.1 (non-fried whole wheat instant noodles fermented with 1.0% yeast and 0.1% lactic acid bacteria).
